# Aortic Arch Variations and Supra-aortic Arterial Tortuosity in Stroke Patients Undergoing Thrombectomy: Retrospective Analysis of 1705 Cases

**DOI:** 10.1007/s00062-022-01181-y

**Published:** 2022-06-13

**Authors:** Maiwand Sidiq, Emilia Scheidecker, Arne Potreck, Ulf Neuberger, Charlotte S. Weyland, Sibu Mundiyanapurath, Martin Bendszus, Markus A. Möhlenbruch, Fatih Seker

**Affiliations:** 1 Neuroradiology, Heidelberg University Hospital, Im Neuenheimer Feld 400, 69120 Heidelberg, Germany; 2 Neurology, Heidelberg University Hospital, Heidelberg, Germany

**Keywords:** Stroke, Mechanical thrombectomy, Aortic arch types, Aortic arch branching patterns, Tortuosity

## Abstract

**Purpose:**

Unfavorable vascular anatomy can impede thrombectomy in patients with acute ischemic stroke. The aim of this study was to determine the prevalence of aortic arch types, aortic arch branching patterns and supra-aortic arterial tortuosity in stroke patients with large vessel occlusion.

**Methods:**

Computed tomography (CT) and magnetic resonance (MR) images of all stroke patients in an institutional thrombectomy registry were retrospectively reviewed. Aortic arch types and branching patterns of all patients were determined. In patients with anterior circulation stroke, the prevalence of tortuosity (elongation, kinking or coiling) of the supra-aortic arteries of the affected side was additionally assessed.

**Results:**

A total of 1705 aortic arches were evaluated. Frequency of aortic arch types I, II and III were 777 (45.6%), 585 (34.3%) and 340 (19.9%), respectively. In 1232 cases (72.3%), there was a normal branching pattern of the aortic arch. The brachiocephalic trunk and the left common carotid artery had a common origin in 258 cases (15.1%). In 209 cases (12.3%), the left common carotid artery arose from the brachiocephalic trunk. Of 1598 analyzed brachiocephalic trunks and/or common carotid arteries, 844 (52.8%) had no vessel tortuosity, 592 (37.0%) had elongation, 155 (9.7%) had kinking, and 7 (0.4%) had coiling. Of 1311 analyzed internal carotid arteries, 471 (35.9%) had no vessel tortuosity, 589 (44.9%) had elongation, 150 (11.4%) had kinking, and 101 (7.7%) had coiling.

**Conclusion:**

With 20%, type III aortic arches are found in a relevant proportion of stroke patients eligible for mechanical thrombectomy. Nearly half of the stroke patients present with supra-aortic arterial tortuosity, mostly arterial elongation.

**Supplementary Information:**

The online version of this article (10.1007/s00062-022-01181-y) contains supplementary material, which is available to authorized users.

## Introduction

Endovascular thrombectomy has been shown to be an effective treatment in acute ischemic stroke due to large vessel occlusion. Nonetheless, successful recanalization is not possible in every case. According to the Highly Effective Reperfusion evaluated in Multiple Endovascular Stroke Trials (HERMES) meta-analysis, successful recanalization could not be achieved in about 29% in the thrombectomy cohort [1]. The Analysis of Pooled Data from Randomized Studies of Thrombectomy More Than 6 Hours After Last Known Well (AURORA) meta-analysis similarly reported that the rate of unsuccessful recanalization in the extended time window was about 20% [2].

One of the reasons for unsuccessful recanalization is difficult arterial access to the target vessel occlusion [3–7]. Especially unfavorable vascular anatomy of the aortic arch and the supra-aortic vessels is associated with a prolonged thrombectomy procedure [8]. Kaymaz et al. reported that supra-aortic vessel tortuosity leads to prolonged time until access to the internal carotid artery is achieved and to lower rates of successful recanalization [9]. Ribo et al. also reported longer procedure times and lower rates of recanalization leading to poor outcome due to difficult arterial access [10].

Unfavorable anatomy is a frequent phenomenon among patients with acute ischemic stroke. In aortic arch types II and III, the supra-aortic arteries arise with a steep angle making it more difficult to navigate catheters [8]. A wide range of prevalences between 35% and 86% have been reported for these aortic arch types [5, 8, 11–14]. Similarly, variant branching patterns, such as the so-called bovine aortic arch can be challenging when trying to achieve access to the left internal carotid artery [8, 9]; however, these branching patterns have been reported with inconsistent nomenclature in the past as pointed out by Layton et al. [15].

Therefore, this present large-scale retrospective study aimed at systematically assessing the prevalence of aortic arch types, branching patterns and supra-aortic arterial tortuosity in stroke patients who underwent thrombectomy at a high-volume comprehensive stroke center over a period of 11 years. These data may help training programs and manufacturers of anatomical flow models and angiography simulators to provide more realistic training scenarios.

## Material and Methods

### Study Design and Setting

This is a retrospective observational single-center analysis at a university hospital in southwest Germany. All stroke patients who were transferred to the angiography suite with the intention to perform endovascular treatment were prospectively collected in an institutional thrombectomy registry. Each thrombectomy case was entered individually in this registry, i.e., a patient undergoing thrombectomy twice due to recurrent large vessel occlusion was entered as two separate cases. Demographic data were collected prospectively. Imaging data (CT or MRI) of all patients, including those that were obtained at referring hospitals, were archived in the institutional PACS.

Institutional review board approval was obtained and informed consent was waived. This manuscript was written according to the Strengthening the Reporting of Observational Studies in Epidemiology statement [16].

### Inclusion and Exclusion Criteria

This study aimed at analyzing two aspects: i) prevalence of aortic arch configurations and branching patterns and ii) prevalence of supra-aortic arterial tortuosity. For these two research questions, the thrombectomy registry was screened for all patients between January 2009 and December 2019 and the following inclusion criteria were chosen.

Regarding i), all patients in the thrombectomy registry with acute ischemic stroke in the anterior and posterior circulation regardless of the occlusion site were included. Patients were excluded if the aortic arch was not depicted on CT angiography (CTA) or MR angiography (MRA) or on other CT or MR images acquired within 3 years before or after endovascular stroke treatment. For instance, a patient might have undergone a chest CT, which can also be used to analyze the aortic arch. This strategy was chosen in order to reduce bias due to unnecessary exclusion of patients.

Regarding ii), all patients with anterior circulation stroke were included. Patients were excluded, if CTA or MRA of the supra-aortic vessels were not available.

### Image Acquisition

Contrast-enhanced CTA and MRA images were obtained by using a variety of CT and MR scanners at stroke centers that participate in a regional stroke network. Acquisition protocols were locally determined and not identical and may have changed over time. In general, a single contrast bolus was given intravenously, followed by a saline flush. Aortic contrast opacification was monitored using bolus tracking. After a certain threshold was achieved, a scan was started from the aortic arch to the head.

### Image Analysis and Parameters

Available CT and MR images were retrieved from the PACS and transferred to a workstation. NUMARIS/4 software (syngo MR B19, Siemens, Erlangen, Germany) was used for three-dimensional image analysis and measurements.

**Fig. 1:**
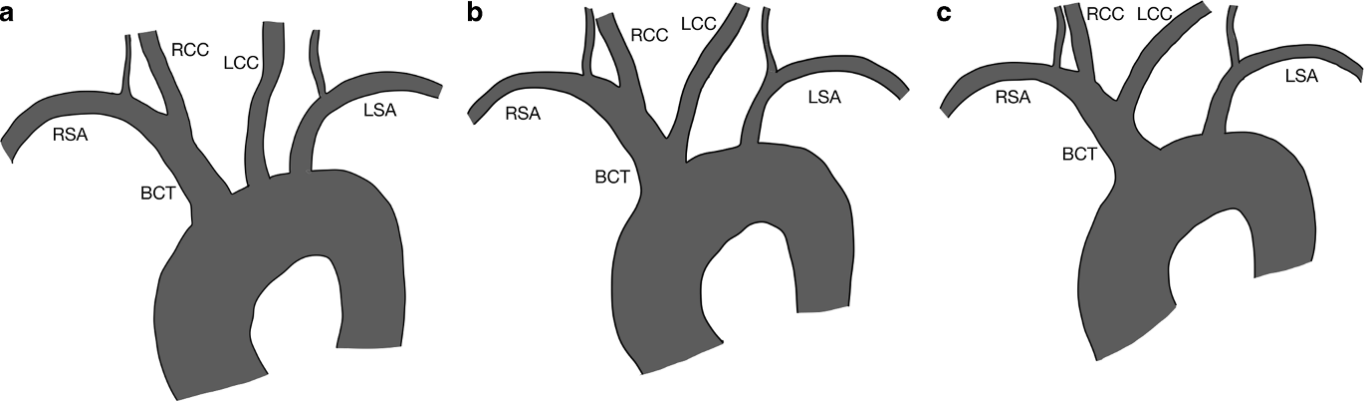
Schematic illustration of aortic arch branching patterns: **a** normal branching pattern (brachiocephalic trunk, left common carotid artery, left subclavian artery), **b** variant with a common origin of the brachiocephalic trunk and the left common carotid artery, and **c** variant in which the left common carotid artery arises from the brachiocephalic trunk. *BCT* brachiocephalic trunk, *LCC* left common carotid artery, *LSA* left subclavian artery, *RCC* right common carotid artery, *RSA* right subclavian artery

Firstly, in all stroke patients, aortic arch types (types I, II and III) were assessed analogously to Snelling et al. [8]: The vertical distance between the most cranial edge of the aortic arch and the distal edge of the branch of the brachiocephalic trunk was measured. If the distance was < 1cm, the aortic arch was termed as type I. If the distance was between 1 and 2 cm, it was termed as type II. If the distance was > 2cm, the aortic arch was termed as type III (Fig. 1).

**Fig. 2:**
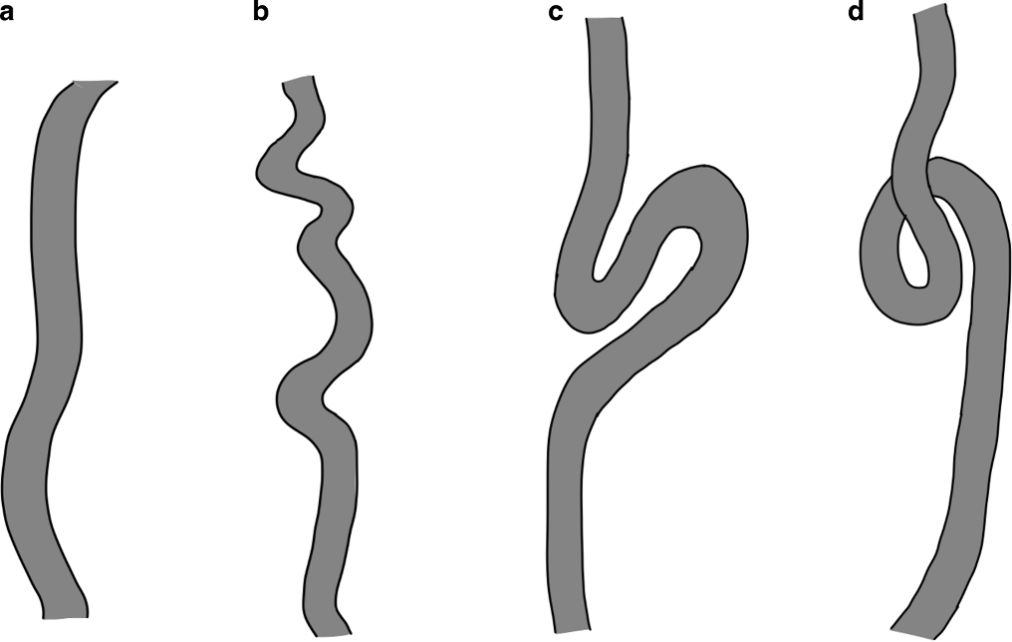
Schematic illustration of different types of supra-aortic arterial tortuosity: **a** straight, **b** elongation, **c** kinking, and **d** coiling

Next, aortic arch branching patterns were also assessed in all stroke patients. Branching patterns were classified as follows: normal branching pattern (brachiocephalic trunk, left common carotid artery, left subclavian artery), a variant with a common origin of the brachiocephalic trunk and the left common carotid artery, a variant in which the left common carotid artery arises from the brachiocephalic trunk or other variants (Fig. 2).

In patients with anterior circulation stroke, the supra-aortic arteries of the affected side (i.e. vascular access for thrombectomy) were analyzed. These arteries were divided into two groups: i) common carotid artery (and brachiocephalic trunk if right-sided) and ii) cervical segment of the internal carotid artery before its entry into the skull base. These two vessel groups were separately classified according to Metz et al. [17] and Weibel and Fields [18] as follows: i) normal with a straight course without or with less tortuosity, ii) S-shaped or C‑shaped elongation or undulation in the course of the artery, iii) kinking with an acute angulation of the vessel, and iv) coiling with an elongation or redundancy of the vessel occurring as an exaggerated S‑shaped curve or in a circular structure [19]. If the internal carotid artery was not sufficiently contrasted due to occlusion, it was deemed as not applicable.

### Statistical Methods

Statistical analysis was performed with R version 3.6.2 and RStudio version 1.2.5033 (RStudio, Boston, MA, USA). Prevalence of aortic arch types, branching patterns and types of supra-aortic arterial tortuosity was assessed. Multivariate logistic regression analysis was performed to identify independent predictors of tortuosity (presence of elongation, kinking or coiling vs. no tortuosity) in i) the brachiocephalic trunk and/or common carotid artery, and in ii) the internal carotid artery. Odds ratios and 95% confidence intervals were estimated. Kruskal-Wallis test was used to compare demographic data among aortic arch types and to compare branching patterns regarding the prevalence of vessel tortuosity (presence of elongation, kinking or coiling vs. no tortuosity). Conover test with Benjamini-Hochberg correction was used for post hoc comparison. A *P*‑value of < 0.05 was considered significant.

## Results

### Demographics

In total, 2017 cases with acute ischemic stroke were screened. Mean patient age was 72.9 ± 13.3 years and *n* = 1029 (51.0%) were female.

### Aortic Arch Types and Branching Patterns

For the analysis of the aortic arch and its branching pattern, 312 out of 2017 cases were excluded, because the aortic arch was not depicted on CTA or MRA (Fig. 1a). In the remaining 1705 cases, the aortic arches and the branching patterns were analyzed.

Frequency of types I, II and III aortic arches were 777 (45.6%), 585 (34.3%) and 340 (19.9%), respectively. In three cases the following variants could not be allocated to these types: in one case, there was a right-sided aortic arch with mirror configuration, i.e. separate common carotid artery and subclavian artery on the right side and a left-sided innominate artery. In another case, there was a right-sided aortic arch with mirror configuration and an asymptomatic chronic occlusion of the left subclavian artery. In one case, there was a right-sided aortic arch with non-mirror configuration and an aberrant left subclavian artery.

Patients with type III aortic arches presented with older age, higher proportion of female gender and more frequently with atrial fibrillation compared to type I and II aortic arches (Table 1).

**Table 1:** Demographics

Characteristics	Type I aortic arch (*n* = 777)	Type II aortic arch (*n* = 585)	Type III aortic arch (*n* = 340)	*P* value^a^	*P* value^b^	*P* value^c^	*P* value^d^
Age, years, mean (SD)	68.4 (14.3)	75.3 (11.1)	79.6 (9.8)	< 0.001	< 0.001	< 0.001	< 0.001
Female, *n* (%)	366 (47.1)	317 (54.2)	201 (59.1)	< 0.001	0.014	< 0.001	0.147
Diabetes, *n* (%)	196 (25.2)	129 (22.1)	66 (19.4)	0.072	–	–	–
Hypertension, *n* (%)	577 (74.3)	440 (75.2)	272 (80.0)	0.072	–	–	–
Coronary heart disease, *n* (%)	192 (24.7)	145 (24.8)	92 (27.1)	0.701	–	–	–
Atrial fibrillation, *n* (%)	282 (36.3)	276 (47.2)	174 (51.2)	< 0.001	< 0.001	< 0.001	0.188
Dyslipidemia, *n* (%)	255 (32.8)	184 (31.5)	103 (30.3)	0.691	–	–	–

*SD* standard deviation

^a^Kruskal-Wallis test between type 1, type 2 and type 3 aortic arches

^b^Post hoc Conover test between type 1 and type 2 aortic arches with Benjamini-Hochberg correction

^c^Post hoc Conover test between type 1 and type 3 aortic arches with Benjamini-Hochberg correction

^d^Post hoc Conover test between type 2 and type 3 aortic arches with Benjamini-Hochberg correction

In 1232 cases (72.3%), there was a normal branching pattern of the aortic arch. The brachiocephalic trunk and the left common carotid artery had a common origin in 258 cases (15.1%). In 209 cases (12.3%), the left common carotid artery arose from the brachiocephalic trunk. In 6 cases, the following variants were observed: 3 of these variants are described above. Furthermore, one patient had an aberrant right subclavian artery (arteria lusoria). In one patient with two endovascular stroke treatments due to recurrent vessel occlusion (i.e. two cases), the left common carotid artery originated from the right common carotid artery.

In none of the cases was a true bovine arch observed [20].

### Tortuosity of the Brachiocephalic Trunk and/or Common Carotid Artery

**Fig. 3:**
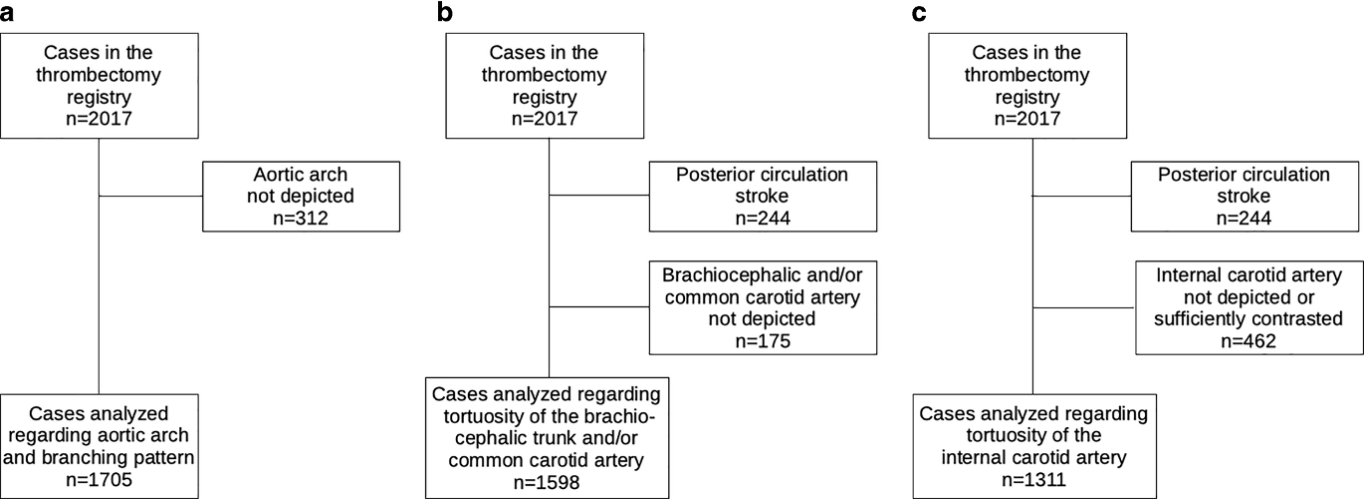
Flow chart of excluded and included cases for the analysis of **a** the aortic arch types and aortic arch branching patterns, **b** the tortuosity of the brachiocephalic trunk and/or common carotid artery, and **c** the tortuosity of the internal carotid artery

For the analysis of supra-aortic vessel tortuosity in the anterior circulation, 2017 cases with acute ischemic stroke were screened and 244 cases with posterior circulation stroke were excluded. Of the remaining 1773 cases, 175 were excluded, because images of the brachiocephalic trunk and/or common carotid artery were not available (Fig. 3b).

Hence, 1598 proximal segments (i.e. brachiocephalic trunk and/or common carotid artery) were analyzed. Of these, 844 (52.8%) had no vessel tortuosity, 592 (37.0%) had elongation, 155 (9.7%) had kinking, and 7 (0.4%) had coiling. In a multivariate analysis, age (adjusted odds ratio 1.05, *P* < 0.001) and hypertension (adjusted odds ratio 1.49, *P* = 0.005) were associated with tortuosity (elongation, kinking or coiling) of the brachiocephalic trunk and/or common carotid artery (Table 2).

**Table 2:** Multivariate analysis on factors associated with supra-aortic vessel tortuosity

	Brachiocephalic trunk and/or common carotid artery (*n* = 1598)		Internal carotid artery (*n* = 1311)	
	Adjusted odds ratio (95% confidence interval)	*P* value	Adjusted odds ratio	*P* value
Age (per year)	1.05 (1.04–1.06)	< 0.001	1.04 (1.02–1.05)	< 0.001
Female	1.19 (0.95–1.48)	0.127	2.10 (1.64–2.69)	< 0.001
Diabetes	0.99 (0.76–1.27)	0.909	0.97 (0.72–1.31)	0.83
Hypertension	1.49 (1.13–1.96)	0.005	1.10 (0.81–1.50)	0.53
Coronary heart disease	1.03 (0.80–1.33)	0.807	1.19 (0.89–1.61)	0.241
Atrial fibrillation	1.14 (0.91–1.42)	0.249	1.24 (0.95–1.60)	0.107
Dyslipidemia	0.80 (0.63–1.02)	0.075	0.74 (0.56–0.98)	0.034

Tortuosity was defined as presence of elongation, kinking or coiling

### Tortuosity of the Internal Carotid Artery

As previously explained 1773 cases with anterior circulation stroke were available. For the analysis of internal carotid artery tortuosity, 462 were excluded, because CTA/MRA of the neck vessels was not available or the internal carotid artery was not assessable due to occlusion and therefore insufficient contrast (Fig. 3c).

Hence, 1311 internal carotid arteries were analyzed. Of these, 471 (35.9%) had no vessel tortuosity, 589 (44.9%) had elongation, 150 (11.4%) had kinking, and 101 (7.7%) had coiling. In a multivariate analysis, age (adjusted odds ratio 1.04, *P* < 0.001) and female gender (adjusted odds ratio 2.1, *P* < 0.001) were associated with tortuosity (elongation, kinking or coiling) of the internal carotid artery. Dyslipidemia was inversely correlated with tortuosity of the internal carotid artery (adjusted odds ratio 0.74, *P* = 0.034) (Table 2).

### Tortuosity Depending on Aortic Arch Branching Pattern

In 1198 cases, both the aortic arch and the entire supra-aortic arteries (i.e. brachiocephalic trunk and/or common carotid artery and the internal carotid artery) were available and sufficiently contrasted for analysis.

In these patients, tortuosity of the brachiocephalic trunk and/or common carotid artery was significantly more prevalent in patients where the left common carotid artery arises from the brachiocephalic trunk compared to those in which both had a common origin (59.7% vs. 41.7%, *P* = 0.027).

Similarly, tortuosity of the internal carotid artery was significantly more prevalent in patients where the left common carotid artery arises from the brachiocephalic trunk compared to those in which both had a common origin (71.1% vs. 54.4%, *P* = 0.007) (Table 3).

**Table 3:** Prevalence of tortuosity depending on aortic arch branching pattern

	Normal branching pattern	Common origin of BT and LCC	LCC arising from BT	*P* value^a^	*P* value^b^	*P* value^c^	*P* value^d^
BT and/or LCC, *n* (%)	439 (51.3)	75 (41.7)	95 (59.7)	0.018	0.15	0.15	0.027
ICA, *n* (%)	559 (65.3)	98 (54.4)	113 (71.1)	0.001	0.016	0.151	0.007

*BT* brachiocephalic trunk, *ICA* internal carotid artery, *LCC* left common carotid artery

^a^Kruskal-Wallis test between type I, type II and type III aortic arches

^b^Post hoc Conover test between type I and type II aortic arches with Benjamini-Hochberg correction

^c^Post hoc Conover test between type I and type III aortic arches with Benjamini-Hochberg correction

^d^Post hoc Conover test between type II and type III aortic arches with Benjamini-Hochberg correction

## Discussion

Supra-aortic vessel tortuosity was first described and categorized by Metz et al. in 1961 and later revised by Weibel and Fields [17, 18]. Anatomical analyses of aortic arch branching patterns even date back to 1933 [21]. Now that endovascular thrombectomy is an evidence-based treatment in acute ischemic stroke due to large vessel occlusion, neurointerventionalists frequently face challenging cases especially in older patients due to unfavorable anatomy [9, 22]. The present large-scale anatomical analysis therefore aimed at systematically assessing the prevalence of aortic arch variants and types as well as supra-aortic arterial tortuosity in stroke patients undergoing thrombectomy.

The reported range of prevalence of type II aortic arches is 20–41% and 15–39% for type III (Supplemental Table 1). Hence, our results of 34.3% for type II and 19.9% for type III aortic arches are within that range. With about 20%, type III aortic arches make up a relevant proportion of thrombectomy cases. Especially in combination with carotid tortuosity, type III aortic arches can be challenging for neurointerventionalists [8].

Older age was associated with aortic arch type III confirming prior studies that the aorta undergoes changes with increasing age [12, 13, 23]. Also, women seem to be more susceptible to this change of aortic geometry compared to men [24]. Some authors such as Demertzis et al. however, reported that there is no correlation with age and gender [11].

Among 1705 analyzed aortic arches in this study, 3 cases with right-sided aortic arches were present. The formation of the aortic arch is indeed a complex process. During the third week of gestation, two aortae are formed which are later connected by several aortic arches [25]. Some arches regress, progress or develop from cranial to caudal forming the final aortic arch and variations [26]. Through regression of the right fourth arch and the right dorsal aorta, the left-sided aortic arch is formed, which is the most common variant. The right-sided aortic arch with mirror configuration develops from regression of the left dorsal aorta and persistence of the right fourth arch [27–29].

Frequently observed branching pattern variants are i) a common origin of the brachiocephalic trunk and the left common carotid artery and ii) the left common carotid artery arising from the brachiocephalic trunk. In our study, these variants had a prevalence of 15.1% and 12.3%, respectively, comparable to results of prior studies (Supplemental Table 2). As pointed out by Layton et al., the umbrella term bovine aortic arch is often falsely used to describe both these variants [15]. In the true bovine anatomy, one single great vessel arises from the aortic arch and gives off the right and left subclavian arteries and then continues as the bicarotid trunk [20]. This variant was not present in any of our cases. Snelling et al. and Rosa et al. developed scores based on the anatomy of the aortic arch and arterial tortuosity to predict procedural time and success of thrombectomy. Both used the term bovine arch and did not differentiate between the two abovementioned variants [8, 22].

Instead of bovine aortic arch, we suggest using other terms such as radix communis (common origin of brachiocephalic trunk and left common carotid artery) and truncus communis (common trunk due to left common carotid artery arising from the brachiocephalic trunk) for these variants as these describe the vascular anatomy more precisely [21]. Interestingly, the truncus communis variant is associated with a higher proportion of vessel tortuosity compared to the radix communis variant. Our data do not offer any explanation for that, though.

The prevalence of supra-aortic arterial tortuosity in our study reflects our observations in the daily practice of thrombectomy. Elongations make up to nearly 40% in our study cohort. Kinking is less frequent with about 10%. Coiling of the internal carotid artery can be seen in nearly 8%. Whether a vessel is kinked or coiled can often only be determined if it is viewed in all dimensions. The prevalence values of some authors differ tremendously, especially regarding kinking and coiling (Supplemental Table 3). This might be related to the study population and how these types of tortuosity were defined [3, 30–33].

Beigelmann et al. and Togay-Isikay et al. stated that carotid tortuosity is a result of alterations in embryological development and not related to age and risk factors [30, 34]; however, our results indicated that older age is associated with supra-aortic vessel tortuosity suggesting that vessels undergo age-related changes over time. Female gender was also associated with tortuosity of the internal carotid artery. This has been similarly reported by Martins et al. and Sacco et al. [32, 35].

Although dyslipidemia is known to be a cardiovascular risk factor, it was inversely correlated with tortuosity of the internal carotid artery in our study. Our data do not offer any explanation for this finding, though. Interestingly, in the abovementioned study by Togay-Isikay et al., hyperlipidemia was slightly less frequent in patients with carotid tortuosity (45.9% vs. 53.1%) [34]. Others such as Del Corso et al. reported significantly higher rates of dyslipidemia in patients with carotid tortuosity [36].

The presented prevalence data may help training programs and manufacturers of anatomical flow models and angiography simulators to provide more realistic training scenarios. Especially fellows who are in the beginning stage of their neurointerventional career, may benefit from a deeper understanding of prevalence and configuration of supra-aortic artery variants and might also benefit from more realistic anatomic models and simulations. The relatively high proportion of type III aortic arches may also encourage device manufacturers to develop catheters, which are especially suited for this type of anatomy.

The main limitation of this study is the retrospective design. It also needs to be stated that this study was performed at a single center in southwest Germany. The majority of our patients were European with only few African and Asian patients. Ethnicity was not assessed in this study, though. The results might have been different if the study had been performed in another country. Besides, approximately 10–15 out of 2026 cases were repeated thrombectomies due to recurrent large vessel occlusion. Each thrombectomy case is individually documented in our registry. This might have led to a slight bias.

## Conclusion

With about 20%, type III aortic arches represent an important proportion in stroke patients eligible for endovascular stroke treatment. Nearly half of the stroke patients presented with supra-aortic arterial tortuosity, mostly arterial elongation.

## Supplementary Information

Supplemental Table 1: Prevalence of Aortic Arch Types
Supplemental Table 2: Prevalance of Aortic Arch Branching Patterns
Supplemental Table 3: Prevalence of Supraaortic Arterial Tortuosity

## Abbreviations

CTA: Computed tomography angiography
MRA: Magnetic resonance angiography
