# Supplementary material for: Aortic Arch Variations and Supra-aortic Arterial Tortuosity in Stroke Patients Undergoing Thrombectomy: Retrospective Analysis of 1705 Cases
Source: Clin Neuroradiol. 2022 Jun 13;33(1):49–56. doi: 10.1007/s00062-022-01181-y (PMC10014756; doi:10.1007/s00062-022-01181-y)
Supplement: Supplementary file 1 — Supplemental Table 1: Prevalence of Aortic Arch Types Supplemental Table 2: Prevalance of Aortic Arch Branching Patterns Supplemental Table 3: Prevalence of Supraaortic Arterial Tortuosity [file 62_2022_1181_MOESM1_ESM.docx]

**Aortic Arch Variations and Supra-aortic Arterial Tortuosity in Stroke Patients Undergoing Thrombectomy: Retrospective Analysis of 1705 Cases**

Maiwand Sidiq,^1^ Emilia Scheidecker,^1^ Arne Potreck, ^1^ Ulf Neuberger,^1^ Charlotte S. Weyland,^1^ Sibu Mundiyanapurath,^2^ Martin Bendszus,^1^ Markus A. Möhlenbruch,^1^ Fatih Seker^1^

^1^ Neuroradiology, Heidelberg University Hospital, Heidelberg, Germany

^2^ Neurology, Heidelberg University Hospital, Heidelberg, Germany

Corresponding author:

Priv.-Doz. Dr. Fatih Seker

ORCID-iD: 0000-0001-6072-0438

Department of Neuroradiology, Heidelberg University Hospital

Im Neuenheimer Feld 400, 69120 Heidelberg, Germany

fatih.seker@med.uni-heidelberg.de

**Supplemental Material**

**Supplemental Table 1** Prevalence of Aortic Arch Types

**Supplemental Table 2** Prevalence of Aortic Arch Branching Patterns

**Supplemental Table 3** Prevalence of Supraaortic Arterial Tortuosity

**Supplemental Table 1** Prevalence of Aortic Arch Types

| **Author** | **No. of patients** | **Patient cohort** | **Methods** | **Results**  **n (%)** | |
| --- | --- | --- | --- | --- | --- |
| Mokin (2020) [1] | 100 | - Stroke patients treated with thrombectomy - Mean age: 72.5 years | CTA | - Type 1: 44 (44%) - Type 2: 41 (41%) - Type 3: 15 (15%) | |
| Snelling (2018) [2] | 61 | - Stroke patients treated with thrombectomy - Median age: 76 years (range 26-93 years) | CTA | - Type 1: 25 (41%) - Type 2: 12 (20%) - Type 3: 24 (39%) | |
| Müller (2017) [3] | 184 | - Patients undergoing carotid stenting (n=97; mean age: 70.5 years) or endarterectomy (n=87; mean age: 71.7 years) | CTA and MRA | - Stenting group:   - Type 1: 37 (38.1)   - Type 2/3: 52(53.6%) | - Endarterectomy group:   - Type 1: 28 (32.2%)   - Type 2/3: 48 (55.2%) |
| Lei Wang (2016) [4] | 2370 | - Heterogenous group - Mean age: 58.1 years (range 18-88 years) | Contrast-enhanced CT | - Type 1: 1384 (58.4%) - Type 3: 752 (31.7%) - Type 3: 234 (9.9%) | |
| Kojima (2016) [5] | 140 | - Patients with suspected cerebrovascular disease - Mean age: 68.0 years | CTA | - Type 1: 20 (14.4%) - Type 2: 30 (21.6%) - Type 3: 89 (64.0%) | |
| Burzotta (2015) [6] | 282 | - Patients undergoing carotid stenting - Mean age: 72 years | Catheter angiography | - Type 1: 184 (65.2%) - Type 2: 66 (23.4%) - Type 3: 32 (11.4%) | |
| Dumont (2014) [7] | 221 | - Patients undergoing carotid stenting - Mean age: 71.6 years (range 34-94 years) | Catheter angiography | - Type 1: 102 (46%) - Type 2: 66 (30%) - Type 3: 53 (24%) | |
| Demertzis (2010) [8] | 92 | - Heterogenous group - Mean age: 69.4 years | CTA | - Type 1: 43 (47%) - Type 2: 33 (36%) - Type 3: 16 (17%) | |
| Lam (2007) [9] | 133 | - Patients undergoing carotid stenting - <80 years (n=96; mean age: 65 years) - ≥80 years (n=37; mean age: 85 years) | Catheter angiography | - <80 years:   - Type 1: 42 (44%)   - Type 2/3: 54 (56%) | - ≥80 years:   - Type 1: 7 (18%)   - Type 2/3: 30 (82%) |

**Supplemental Table 2** Prevalence of Aortic Arch Branching Patterns

| **Author** | **No. of patients** | **Patient cohort** | **Methods** | **Results**  **n (%)** |
| --- | --- | --- | --- | --- |
| Mokin (2020) [1] | 100 | - Stroke patients treated with thrombectomy - Mean age: 72.5 years | CTA | - CO + CTr: 25 (25%) |
| Syperek (2019) [10] | 474 | - 152 stroke patients (mean age: 72 years; range 40-88 years) - 322 healthy patients (mean age: 65.8 years; range 29-94 years) | Contrast-enhanced CT and CTA | - Stroke group:   - NBP: 105 (69.1%)   - CO: 23 (15.1%)   - CTr: 16 (10.5%)   - Others: 8 (5.3%) - Control group:   - NBP: 251 (78.0%)   - CO: 39 (12.1%)   - CTr: 16 (5.0%)   - Others: 16 (5.0%) |
| Snelling (2018) [2] | 61 | - Stroke patients treated with thrombectomy - Median age: 76 years (range 26-93 years) | CTA | - CO + CTr: 10 (16%) |
| Müller (2017) [3] | 184 | - Patients undergoing carotid stenting (n=97; mean age: 70.5 years) or endarterectomy (n=87; mean age: 71.7 years) | CTA and MRA | - Stenting: 11 CTr (11.3%) - Endarterectomy: 10 CTr (11.5%) |
| Kaymaz (2017) [11] | 76 | - Stroke patients treated with thrombectomy - NBP (mean age: 68.7 years) and CO + CTr (mean age: 70.6 years) | CTA | - NBP: 42 (55.3%) - CO + CTr: 34 (44.7%) |
| Feiz (2017) [12] | 248 | - Stroke patients treated with thrombectomy - Control group of 808 patients under 60 years - Mean age of all: 51.5 years (range 4 months - 93 years) | CTA | - Stroke cohort:   - NBP: 208 (83.9%)   - CTr: 22(8.9%)   - Others: 20 (8.1%) - Control group:   - NBP: 696 (86.1%)   - CTr: 67 (8.3%)   - Others: 47 (5.8%) |

| Mustafa (2016) [13] | 500 | - Heterogenous group - Mean age: 54.0 years (range 2-92 years) | CTA | - NBP: 306 (61.2%) - CO: 156 (31.2%) - Others: 38 (7.6%) |
| --- | --- | --- | --- | --- |
| Lei Wang (2016) [4] | 2370 | - Heterogenous group - Mean age: 58.1 years (range 18-88 years) | Contrast-enhanced CT | - NBP: 1985 (83.8%) - CO: 245 (10.3%) - Others: 140 (5.9%) |
| Kojima (2016) [5] | 140 | - Patients with suspected cerebrovascular disease - Mean age: 68.0 years | CTA | - NBP: 118 (84.3%) - CO: 20 (14.2%) - Others: 2 (1.4%) |
| Dumfarth (2015) [14] | 556 | - Patients with thoracic aortic disease (mean age: 58.9 years; range 6-85 years) compared to a historical group of 4617 patients | CT and MRI | - Thoracic aortic disease group:   - NBP: 370 (66.5%)   - CO + CTr: 141 (25.4%)   - Others: 45 (8.1%) - Historical group:   - NBP: 3740 (81%)   - CO + CTr: 646 (14%)   - Others: 185 (4%) |
| Burzotta (2015) [6] | 282 | - Patients undergoing carotid stenting - Mean age: 72 years | Catheter angiography | - NBP: 224 (79.4%) - CO: 41 (14.5%) - CTr: 17 (6.0%) |
| Dumont (2014) [7] | 221 | - Patients undergoing carotid stenting - Mean age: 71.6 years (range 34-94 years) | Catheter angiography | - CO + CTr: 51 (23%) |
| Karacan (2014) [15] | 1000 | - Heterogenous group with a normal left-sided aortic arch - Mean age: 56 years (range 17-94 years) | CTA | - NBP: 792 (79.2%) - CO: 153 (15.3%) - Others: 55 (5.5%) |
| Rea (2014) [16] | 1359 | - Heterogenous group - Median age: 48.3 years (range 17-89 years) | CTA | - NBP: 965 (71%) - CO: 302 (22%) - CTr: 21 (1.5%) - Others: 66 (4.9%) |
| Lale (2014) [17] | 881 | - Heterogenous group - Mean age: 62 years (range 19-93 years) | CTA | - NBP: 770 (87.4%) - CTr: 64 (7.2%) - Others: 47 (5.3%) |

| Reinshagen (2014) [18] | 2033 | - Children undergoing heart catheterization | Catheter angiography | - NBP: 1723 (84.8%) - CO: 230 (11.3%) - CTr: 80 (3.9%) |
| --- | --- | --- | --- | --- |
| Ergun (2013) [19] | 1001 | - Heterogenous group - Mean age: 60.0 years (range 18-88 years) | CTA | - NBP: 853 (85.2%) - CO + CTr: 78 (7.8%) - Others: 70 (7.0%) |
| Budhiraja (2013) [20] | 52 | - Dissection study of an Indian population | Cadaver study | - NBP: 33 (63.5%) - CO: 11 (21.2%) - Others: 8 (15.3%) |
| Uchino (2013) [21] | 2352 | - Patients with cerebrovascular disease - Mean age: 63.2 years (range 8-97 years) | CTA | - NBP: 2070 (88.0%) - CO: 130 (5.5%) - CTr 141 (6.0%) - Others: 11 (0.47%) |
| Vučurević (2013) [22] | 1266 | - Heterogenous group - Mean age: 68 years (males) and 65 years (females) | CT, CTA and catheter angiography | - NBP: 946 (74.7%) - CO: 86 (6.8%) - CTr: 111 (8.8%) - Others: 123 (9.7%) |
| Celikyay (2013) [23] | 1136 | - Heterogenous group | Contrast-enhanced CT and CTA | - NBP: 845 (74.4%) - CO + CTr: 247 (21.7%) - Others: 44 (3.9%) |
| Hornick (2012) [24] | 612 | - Patients with thoracic aortic aneurysm, dissection or rupture - Control group without thoracic aortic disease (n=844, mean age: 55.7 years) | CT and MRI | - Pathological cohort:   - CO: 108 (17.6%)   - CTr: 53 (8.7%) - Control group:   - CO + CTr: 138 (16.4%) |
| Malone (2012) [25] | 191 | - Patients with aortic dilatation (mean age: 68.5 years) - Control group of 391 patients (mean age: 59.6 years) | CT and MRI | - Pathological cohort:   - CO + CTr: 50 (26.2%) - Control group:   - CO + CTr: 80 (20.5%) |

| Müller (2011) [26] | 2033 | - Heterogenous group; mean age: 61 years - Group A: n=429 (age range 3 months – 49 years) - Group B: n=1604 (age range 50 – 94 years) | CT and CTA | - Group A:   - NBP: 371 (86.5%)   - CTr: 31 (7.2%)   - Others: 27 (6.3%) - Group B:   - NBP: 1393 (86.8%)   - CTr 132(8.2%)   - Others: 79 (4.9%) |
| --- | --- | --- | --- | --- |
| Piyavisetpat (2011) [27] | 687 | - Heterogenous group, minimum age 15 years (range 18-94 years) | Contrast-enhanced CT | - NBP: 611 (88.9%) - CO: 34 (5.0%) - CTr: 7 (1.0%) - Others: 35 (5.1%) |
| Jakanani (2010) [28] | 861 | - Heterogenous group | CT unenhanced and with contrast medium | - NBP: 643 (74.7%) - CO: 197 (22.9%) - Others: 21 (2.4%) |
| Demertzis (2010) [8] | 92 | - Heterogenous group - Mean age: 69.4 years | CTA | - NBP: 82 (89.1%) - CO: 8 (8.7%) - Others: 2 (2.2%) |
| Ogeng’o (2010) [29] | 113 | - Dissection study of Kenyan cadavers | Cadaver study | - NBP: 76 (67.3%) - CO: 32 (28.3%) - Others: 5 (4.4%) |
| Natsis (2009) [30] | 633 | - Heterogenous group (age range 19-79 years) | Catheter angiography | - NBP: 527 (83%) - CO + CTr: 96 (15%) - Others: 10 (1.6%) |
| Berko (2009) [31] | 1000 | - Patients with suspected pulmonary embolism or aortic dissection - Median age: 53 years (range 19-97 years) | CTA | - NBP: 665 (66.0%) - CO + CTr: 274 (27.4%) - Others: 61 (6.1%) |
| Faggioli (2007) [32] | 214 | - Patients undergoing carotid stenting - NBP (mean age: 76.1 years) and arch anomalies (mean age: 79.9 years) | Catheter angiography | - NBP: 189 (88.4%) - CO + CTr: 22 (10.2%) - Others: 3 (1.4%) |
| Makhanya (2004) [33] | 60 | - 60 randomly selected patients on whom arch aortograms were performed | Catheter angiography | - NBP: 39 (65.0%) - CO + CTr: 18 (28.3%) - Others: 3 (5.0%) |

| Nelson (2001) [34] | 193 | - Heterogenous group | Cadaver study | - NBP: 182 (94.3%) - CO: 2 (1.0%) - Others: 9 (4.7%) |
| --- | --- | --- | --- | --- |
| Reppert (1993) [35] | 705 | - Arteriograms taken due to various reasons | Catheter angiography | - NBP: 629 (89.2%) - CO: 53 (7.5%) - CTr: 18 (2.6%) - Others: 5 (0.7%) |
| Liechty (1957) [36] | 1000 | - Dissection study | Cadaver study | - NBP: 649 (64.9%) - CO: 284 (28.4%) - Others: 67 (6.7%) |
| De Garis (1933) [37] | 314 | - White (n=111) and African American (n=203) cadavers | Cadaver study | - White:   - NBP: 86 (77.5%)   - CO: 9 (8.1%)   - CT: 6 (5.4%)   - Others: 10 (9.0%) - African American:   - NBP: 97 (47.8%)   - CO: 51 (25.1%)   - CTr: 21 (10.3%)   - Others: 34 (16.8%) |

Abbreviations:

- Normal branching pattern (NBP): Brachiocephalic trunk, left common carotid artery, left subclavian artery
- Common origin (CO): Common origin of the brachiocephalic trunk and the left common carotid artery
- CTA: CT angiography
- Common trunk (CTr): Left common carotid artery arising from the brachiocephalic trunk
- MRA: MR angiography

**Supplemental Table 3** Prevalence of Supraaortic Arterial Tortuosity

| **Author** | **No. of patients** | **Patient cohort** | **Methods** | **Results** |
| --- | --- | --- | --- | --- |
| Leker (2020) [38] | 302 | - Stroke patients treated with thrombectomy - Mean age: 70 years | CTA of cervical ICA:   - Grade 1: normal vessel or mild tortuosity. - Grade 2: severe tortuosity, coiling, kinking. | - Grade 1: 159 patients (53%) - Grade 2: 143 patients (47%) |
| Di Pino (2020) [39] | 2856 | - Heterogenous cohort with sick and healthy people - Mean age: 58 years (range 0-96 years) | Ultrasound of cervical ICA; bilateral | - Coiling or kinking: 284 patients (9.9%) - 74 of 284 patients (26.1%) with bilateral coiling/kinking - 357 extracranial ICA anomalies:   - Kinking: 321 arteries (89.9%)   - Coiling: 36 arteries (10.1%) |
| Benson (2020) [40] | 120 | - Stroke patients undergoing thrombectomy - Mean age: 68.3 years | CTA of cervical ICA; bilateral | - 47 patients (39.2%) with any form of tortuosity on one or both sides:   - Kinks: 28 (23.3%)   - Loops: 20 (16.7%)   - Coils: 8 (6.7%) |
| Snelling (2018) [2] | 61 | - Stroke patients treated with thrombectomy - Median age: 76 years (range 26-93 years) | CTA of ICA | - ICA anatomy of all patients:   - Tortuosity: 20 patients (33%)   - Kinking: 22 patients (36%)   - Coiling: 3 patients (5%) |
| Martins (2018) [41] | 19804 | - Neurological patients over 25 years - Mean age: 66 years (range 25-99 years) | Ultrasound of proximal common carotid artery, distal ICA and external carotid artery; bilateral | - 2678 patients (13.5%) with morphological changes:   - Kinking: 2147 patients (80.2%)   - Coiling: 426 patients (15.9%)   - Looping: 24 patients (0.9%)   - Concurrent: 81 patients (3.0%) |
| Gocmen (2017) [42] | 100 | - Stroke patients treated with thrombolysis - Mean age: 69 years | CTA of cervical carotid artery; bilateral | - ICA course ipsilateral / contralateral:   - Tortuosity: 45 arteries (45%) / 44 (44%)   - Kinking: 39 arteries (39%) / 39 (39%)   - Coiling: 4 arteries (4%) / 6 (6%) |
| Pfeiffer (2016) [43] | 125 | - Adults with highly aberrant course of the extracranial ICA - Mean age: 67.2 years (range 18-97 years) | MR and CT images with and without contrast medium and CTA/MRA of cervical ICA; bilateral | - 231 aberrant ICA courses:   - Tortuosity: 99 arteries (42.9%)   - Kinking: 104 arteries (45.0%)   - Coiling: 28 arteries (12.1%) |
| Nagata (2016) [44] | 148 | - Patients treated in the Department of Oral and Maxillofacial Surgery - Median age: 63.9 years (range 13-88 years) | Contrast-enhanced CT | - 296 arteries:   - Tortuosity: 254 arteries (85.8%)   - Coiling: 9 arteries (3.0%)   - Kinking: 3 arteries (1.0%)   - Occlusion: 2 arteries (0.7%) |
| Saba (2015) [45] | 124 | - Patients with ICA dissection - Median age: 57 years | CT and MR images of ICA; bilateral | - 248 arteries:   - Elongation: 55 arteries (22.17%)   - Kinking: 48 arteries (19.35%)   - Coiling: 22 arteries (8.9%) |
| Yu (2015) [46] | 702 | - Heterogenous group ≥50 years - Mean age: 65.5 years | CTA of extracranial carotid artery; bilateral | - 1404 carotid arteries:   - Tortuosity: 617 arteries (43.9%)   - Kinking: 355 arteries (25.3%)   - Coiling: 15 arteries (1.1%) |
| Saba (2010) [47] | 153 | - Patients with stenosis or previously described TIA or stroke - Mean age: 67.3 years (range 41-83 years) | CTA of carotid arteries; bilateral | - 306 carotid arteries:   - Kinking: 37 arteries (12.1%)   - Coiling: 20 arteries (6.5%) |
| Beigelman (2010) [48] | 885 | - Heterogenous groups - Group 1 (healthy, n=245) up to 15 years (mean age: 6 years) - Group 2 (n=640) underwent ultrasound (age range 16-90 years) due to atherosclerosis (mean age: 57 years) | Ultrasound of carotid arteries | - Group 1:   - Coiling: 10 patients (4%)   - Kinking: 67 patients (27%) - Group 2:   - Coiling: 19 patients (3%)   - Kinking: 143 patients (22%) |

| Sacco (2007) [49] | 1217 | - Heterogenous group - Mean age: 62.7 years | Ultrasound of ICA; bilateral | - 319 patients (26.2%) with 437 ICA morphologic variations:   - Tortuosity: 195 arteries (44.6%)   - Kinking: 236 arteries (54.0%)   - Coiling: 6 arteries (1.4%) |
| --- | --- | --- | --- | --- |
| Togay-Işikay (2005) [50] | 345 | - Vascular and neurological patients - Mean age: 67 years (with carotid abnormalities) and 64 years (without abnormalities) | Ultrasound of ICA; bilateral | - 85 patients (24.6%) with carotid abnormalities in 126 arteries:   - Tortuosity: 48 arteries (38%)   - Kinking: 71 arteries (56%)   - Coiling: 7 arteries (6%) |
| Paulsen (2000) [51] | 164 | - Preparations from body donors (n=282) - Age range 52-98 years - Another 100 head and neck halves randomly selected to relate variations in ICA course to age | Cadaver study of ICA; bilateral | - ICA course in specimens:   - Curved: 74 patients (26.2%)   - Kinking: 12 patients (4.2%)   - Coiling: 5 patients (1.8%) - Control group:   - Curved: 25 patients (25%)   - Kinking: 4 patients (4%)   - Coiling: 2 patients (2%) |
| Pellegrino (1998) [52] | 1220 | - Patients with extracranial carotid dolichoarterial disease - Age range 25-89 years | Ultrasound of extracranial carotid artery; bilateral | - 316 patients (25.9%) with carotid artery dolichoarterial disease:   - Tortuosity: 109 patients (8.9%)   - Kinking: 171 patients (14%)   - Coiling: 36 patients (2.9%) |
| Del Corso (1998) [53] | 469 | - Cardiovascular disease or neurologic or geriatric patients - Mean age: 66.4 years (range 24-89 years) | Ultrasound of carotid artery; bilateral | - 272 patients (58%) with 479 abnormalities:   - Tortuosities: 104 in 73 patients (15.6%)   - Kinks: 262 in 188 patients (40.1%)   - Coils: 113 in 88 patients (18.8%) |
| Macchi (1997) [54] | 100 | - Healthy probands - Mean age: 67.5 years (range 18-98 years) | Ultrasound of the supra-aortic trunks (brachioradialis, subclavian, common carotid, internal carotid, external carotid); bilateral | - Kinking in 38 patients (38%):   - Bilateral: 15 patients   - Left: 12 patients   - Right: 11 patients |

| Oliviero (1997) [55] | 235 | - Patients with hypertension (n=130; mean age: 58 years) or diabetes (n=105; mean age: 59 years) - Healthy control group (n=50; mean age: 60 years) | Ultrasound of extracranial carotid arteries | - Hypertensives / diabetics / healthy:   - Kinking: 16 patients (12.3%) / 2 (2%) / 2 (4%)   - Coiling: 3 patients (2.3%) / 0 / 0 |
| --- | --- | --- | --- | --- |
| Borioni (1994) [56] | 653 | - Mostly patients with coronary artery disease - Mean age: 58.3 years | Ultrasound of ICA; bilateral | - Incidence of all patients:   - Tortuosity: 9 patients (1.4%)   - Kinking: 28 patients (4.3%) |
| Barbour (1994) [57] | 121 | - 13 patients with ICA dissection (mean age: 54.3 years) - 108 patients undergoing catheter angiography as a control group (mean age: 68.0 years) - Age range 39-89 years | Catheter angiography of ICA; bilateral | - Dissection group:   - 8/13 patients (62%) and 13/20 vessels with redundancies (65%): 2 loops, 9 coils, 2 kinks - Control group:   - 20/108 patients (19%) and 22/187 (12%) vessels with redundancies: 3 loops, 10 coils, 9 kinks |
| Metz (1961) [58] | 1000 | - Heterogenous group of patients undergoing cerebral angiography | Catheter angiography of ICA | - 161 cases with kinks (16%) |

Abbreviations:

- ICA: internal carotid artery
- CTA: CT angiography
- MRA: MR angiography

**References**

1. Mokin M, Waqas M, Chin F, Rai H, Senko J, Sparks A, Ducharme RW, Springer M, Borlongan CV, Levy EI, Ionita C, Siddiqui AH. Semi-automated measurement of vascular tortuosity and its implications for mechanical thrombectomy performance. Neuroradiology. 2020. <https://doi.org/10.1007/s00234-020-02525-6>.

2. Snelling BM, Sur S, Shah SS, Chen S, Menaker SA, McCarthy DJ, Yavagal DR, Peterson EC, Starke RM. Unfavorable Vascular Anatomy Is Associated with Increased Revascularization Time and Worse Outcome in Anterior Circulation Thrombectomy. World Neurosurgery. 2018;120:e976-e83. <https://doi.org/https://doi.org/10.1016/j.wneu.2018.08.207>.

3. Müller MD, Ahlhelm FJ, von Hessling A, Doig D, Nederkoorn PJ, Macdonald S, Lyrer PA, van der Lugt A, Hendrikse J, Stippich C, van der Worp HB, Richards T, Brown MM, Engelter ST, Bonati LH. Vascular Anatomy Predicts the Risk of Cerebral Ischemia in Patients Randomized to Carotid Stenting Versus Endarterectomy. Stroke. 2017;48(5):1285-92. <https://doi.org/10.1161/strokeaha.116.014612>.

4. Wang L, Zhang J, Xin S. Morphologic features of the aortic arch and its branches in the adult Chinese population. J Vasc Surg. 2016;64(6):1602-8.e1. <https://doi.org/10.1016/j.jvs.2016.05.092>.

5. Kojima A, Saga I. Effect of aging on the configurational change of the aortic arch. Geriatric Care. 2016;2. <https://doi.org/10.4081/gc.2016.5720>.

6. Burzotta F, Nerla R, Pirozzolo G, Aurigemma C, Niccoli G, Leone AM, Saffioti S, Crea F, Trani C. Clinical and procedural impact of aortic arch anatomic variants in carotid stenting procedures. Catheter Cardiovasc Interv. 2015;86(3):480-9. <https://doi.org/10.1002/ccd.25947>.

7. Dumont TM, Mokin M, Wach MM, Drummond PS, Siddiqui AH, Levy EI. Understanding risk factors for perioperative ischemic events with carotid stenting: is patient age over 80 years or is unfavorable arch anatomy to blame? J Neurointerv Surg. 2014;6. <https://doi.org/10.1136/neurintsurg-2013-010721>.

8. Demertzis S, Hurni S, Stalder M, Gahl B, Herrmann G, Van den Berg J. Aortic arch morphometry in living humans. J Anat. 2010;217(5):588-96. <https://doi.org/10.1111/j.1469-7580.2010.01297.x>.

9. Lam RC, Lin SC, DeRubertis B, Hynecek R, Kent KC, Faries PL. The impact of increasing age on anatomic factors affecting carotid angioplasty and stenting. J Vasc Surg. 2007;45(5):875-80. <https://doi.org/10.1016/j.jvs.2006.12.059>.

10. Syperek A, Angermaier A, Kromrey M-L, Hosten N, Kirsch M. The so-called “bovine aortic arch”: a possible biomarker for embolic strokes? Neuroradiology. 2019;61(10):1165-72. <https://doi.org/10.1007/s00234-019-02264-3>.

11. Kaymaz ZO, Nikoubashman O, Brockmann MA, Wiesmann M, Brockmann C. Influence of carotid tortuosity on internal carotid artery access time in the treatment of acute ischemic stroke. Interv Neuroradiol. 2017;23(6):583-8. <https://doi.org/10.1177/1591019917729364>.

12. Feiz M NO, Müller M, Schiefer J, Brockmann C, Reich A, et al. Frequency of Aortic Arch Variants in Patients with Large Vessel Stroke in the Anterior Circulation. Austin J Cerebrovasc Dis & Stroke. 2017;4(1):1051.

13. Mustafa AG, Allouh MZ, Ghaida JHA, Al-Omari MmH, Mahmoud WA. Branching patterns of the aortic arch: a computed tomography angiography-based study. Surgical and Radiologic Anatomy. 2017;39(3):235-42. <https://doi.org/10.1007/s00276-016-1720-z>.

14. Dumfarth J, Chou AS, Ziganshin BA, Bhandari R, Peterss S, Tranquilli M, Mojibian H, Fang H, Rizzo JA, Elefteriades JA. Atypical aortic arch branching variants: A novel marker for thoracic aortic disease. J Thorac Cardiovasc Surg. 2015;149(6):1586-92. <https://doi.org/10.1016/j.jtcvs.2015.02.019>.

15. Karacan A, Türkvatan A, Karacan K. Anatomical variations of aortic arch branching: evaluation with computed tomographic angiography. Cardiol Young. 2014;24(3):485-93. <https://doi.org/10.1017/s1047951113000656>.

16. Rea G, Valente T, Iaselli F, Urraro F, Izzo A, Sica G, Muto M, Scaglione M, Muto M, Rotondo A. Multi-detector computed tomography in the evaluation of variants and anomalies of aortic arch and its branching pattern. Italian journal of anatomy and embryology = Archivio italiano di anatomia ed embriologia. 2014;119:180-92. <https://doi.org/10.13128/IJAE-15541>.

17. Lale P, Toprak U, Yagız G, Kaya T, Uyanık SA. Variations in the Branching Pattern of the Aortic Arch Detected with Computerized Tomography Angiography. Advances in Radiology. 2014;2014:969728. <https://doi.org/10.1155/2014/969728>.

18. Reinshagen L, Vodiskar J, Mühler E, Hövels-Gürich HH, Vazquez-Jimenez JF. Bicarotid Trunk: How Much Is “Not Uncommon”? The Annals of Thoracic Surgery. 2014;97(3):945-9. <https://doi.org/https://doi.org/10.1016/j.athoracsur.2013.12.014>.

19. Ergun E, Şimşek B, Koşar PN, Yılmaz BK, Turgut AT. Anatomical variations in branching pattern of arcus aorta: 64-slice CTA appearance. Surg Radiol Anat. 2013;35(6):503-9. <https://doi.org/10.1007/s00276-012-1063-3>.

20. Budhiraja V, Rastogi R, Jain V, Bankwar V, Raghuwanshi S. Anatomical Variations in the Branching Pattern of Human Aortic Arch: A Cadaveric Study from Central India. ISRN Anatomy. 2013;2013:828969. <https://doi.org/10.5402/2013/828969>.

21. Uchino A, Saito N, Okada Y, Kozawa E, Nishi N, Mizukoshi W, Nakajima R, Takahashi M, Watanabe Y. Variation of the origin of the left common carotid artery diagnosed by CT angiography. Surg Radiol Anat. 2013;35(4):339-42. <https://doi.org/10.1007/s00276-012-1038-4>.

22. Vučurević G, Marinković S, Puškaš L, Kovačević I, Tanasković S, Radak D, Ilić A. Anatomy and radiology of the variations of aortic arch branches in 1,266 patients. Folia Morphol (Warsz). 2013;72(2):113-22. <https://doi.org/10.5603/fm.2013.0019>.

23. Celikyay ZR, Koner AE, Celikyay F, Denız C, Acu B, Firat MM. Frequency and imaging findings of variations in human aortic arch anatomy based on multidetector computed tomography data. Clin Imaging. 2013;37(6):1011-9. <https://doi.org/10.1016/j.clinimag.2013.07.008>.

24. Hornick M, Moomiaie R, Mojibian H, Ziganshin B, Almuwaqqat Z, Lee ES, Rizzo JA, Tranquilli M, Elefteriades JA. 'Bovine' aortic arch - a marker for thoracic aortic disease. Cardiology. 2012;123(2):116-24. <https://doi.org/10.1159/000342071>.

25. Malone CD, Urbania TH, Crook SE, Hope MD. Bovine aortic arch: a novel association with thoracic aortic dilation. Clin Radiol. 2012;67(1):28-31. <https://doi.org/10.1016/j.crad.2011.04.004>.

26. Müller M, Schmitz BL, Pauls S, Schick M, Röhrer S, Kapapa T, Schlötzer W. Variations of the aortic arch - a study on the most common branching patterns. Acta Radiol. 2011;52(7):738-42. <https://doi.org/10.1258/ar.2011.110013>.

27. Piyavisetpat N, Thaksinawisut P, Tumkosit M. Aortic arch branches' variations detected on chest CT. Asian Biomedicine. 2011;5. <https://doi.org/10.5372/1905-7415.0506.106>.

28. Jakanani GC, Adair W. Frequency of variations in aortic arch anatomy depicted on multidetector CT. Clin Radiol. 2010;65(6):481-7. <https://doi.org/10.1016/j.crad.2010.02.003>.

29. Ogeng'o JA, Olabu BO, Gatonga PM, Munguti JK. Branching pattern of aortic arch in a kenyan population. Journal of Morphological Sciences. 2010;27:51-5.

30. Natsis KI, Tsitouridis IA, Didagelos MV, Fillipidis AA, Vlasis KG, Tsikaras PD. Anatomical variations in the branches of the human aortic arch in 633 angiographies: clinical significance and literature review. Surg Radiol Anat. 2009;31(5):319-23. <https://doi.org/10.1007/s00276-008-0442-2>.

31. Berko NS, Jain VR, Godelman A, Stein EG, Ghosh S, Haramati LB. Variants and anomalies of thoracic vasculature on computed tomographic angiography in adults. J Comput Assist Tomogr. 2009;33(4):523-8. <https://doi.org/10.1097/RCT.0b013e3181888343>.

32. Faggioli GL, Ferri M, Freyrie A, Gargiulo M, Fratesi F, Rossi C, Manzoli L, Stella A. Aortic arch anomalies are associated with increased risk of neurological events in carotid stent procedures. Eur J Vasc Endovasc Surg. 2007;33(4):436-41. <https://doi.org/10.1016/j.ejvs.2006.11.026>.

33. Makhanya NZ, Mamogale RT, Khan N. Variants of the left aortic arch branches. 2004. 2004;8(4). <https://doi.org/10.4102/sajr.v8i4.102>.

34. Nelson ML, Sparks CD. Unusual aortic arch variation: Distal origin of common carotid arteries. Clinical Anatomy. 2001;14(1):62-5. <https://doi.org/10.1002/1098-2353(200101)14:1><62::Aid-ca1012>3.0.Co;2-#.

35. Reppert MK, Lundgren EC, Dibos LA, Deshmukh N. Variations in Aortic Arch Branch Vessel Anatomy as Seen by Aortography. Vascular Surgery. 1993;27(2):89-93. <https://doi.org/10.1177/153857449302700202>.

36. Liechty JD, Shields TW, Anson BJ. Variations pertaining to the aortic arches and their branches; with comments on surgically important types. Q Bull Northwest Univ Med Sch. 1957;31(2):136-43.

37. De Garis CF, Black IH, Riemenschneider EA. Patterns of the Aortic Arch in American White and Negro Stocks, with Comparative Notes on Certain Other Mammals. Journal of anatomy. 1933;67(Pt 4):599-619.

38. Leker RR, Kasner SE, El Hasan HA, Sacagiu T, Honig A, Gomori JM, Guan S, Choudhry O, Hurst RW, Kung D, Pukenas B, Sedora-Roman N, Ramchand P, Cohen JE. Impact of carotid tortuosity on outcome after endovascular thrombectomy. Neurol Sci. 2021;42(6):2347-51. <https://doi.org/10.1007/s10072-020-04813-8>.

39. Di Pino L, Franchina AG, Costa S, Gangi S, Strano F, Ragusa M, Costanzo L, Tamburino C, Capodanno D. Prevalence and morphological changes of carotid kinking and coiling in growth: an echo-color Doppler study of 2856 subjects between aged 0 to 96 years. The International Journal of Cardiovascular Imaging. 2020. <https://doi.org/10.1007/s10554-020-02014-0>.

40. Benson JC, Brinjikji W, Messina SA, Lanzino G, Kallmes DF. Cervical internal carotid artery tortuosity: A morphologic analysis of patients with acute ischemic stroke. Interv Neuroradiol. 2020;26(2):216-21. <https://doi.org/10.1177/1591019919891295>.

41. Martins HFG, Mayer A, Batista P, Soares F, Almeida V, Pedro AJ, Oliveira V. Morphological changes of the internal carotid artery: prevalence and characteristics. A clinical and ultrasonographic study in a series of 19 804 patients over 25 years old. European Journal of Neurology. 2018;25(1):171-7. <https://doi.org/10.1111/ene.13491>.

42. Gocmen R, Arsava EM, Oguz KK, Topcuoglu MA. Intravenous Thrombolysis for Acute Ischemic Stroke in Patients with Cervicocephalic Dolichoarteriopathy. Journal of Stroke and Cerebrovascular Diseases. 2017;26(11):2579-86. <https://doi.org/https://doi.org/10.1016/j.jstrokecerebrovasdis.2017.06.001>.

43. Pfeiffer J, Becker C, Ridder GJ. Aberrant extracranial internal carotid arteries: New insights, implications, and demand for a clinical grading system. Head Neck. 2016;38 Suppl 1:E687-93. <https://doi.org/10.1002/hed.24071>.

44. Nagata T, Masumoto K, Hayashi Y, Watanabe Y, Kato Y, Katou F. Three-dimensional computed tomographic analysis of variations of the carotid artery. Journal of Cranio-Maxillofacial Surgery. 2016;44(6):734-42. <https://doi.org/https://doi.org/10.1016/j.jcms.2016.02.011>.

45. Saba L, Argiolas GM, Sumer S, Siotto P, Raz E, Sanfilippo R, Montisci R, Piga M, Wintermark M. Association between internal carotid artery dissection and arterial tortuosity. Neuroradiology. 2015;57(2):149-53. <https://doi.org/10.1007/s00234-014-1436-x>.

46. Yu K, Zhong T, Li L, Wang J, Chen Y, Zhou H. Significant Association between Carotid Artery Kinking and Leukoaraiosis in Middle-Aged and Elderly Chinese Patients. J Stroke Cerebrovasc Dis. 2015;24(5):1025-31. <https://doi.org/10.1016/j.jstrokecerebrovasdis.2014.12.030>.

47. Saba L, Mallarini G. Correlation between kinking and coiling of the carotid arteries as assessed using MDCTA with symptoms and degree of stenosis. Clinical Radiology. 2010;65(9):729-34. <https://doi.org/https://doi.org/10.1016/j.crad.2010.04.015>.

48. Beigelman R, Izaguirre AM, Robles M, Grana DR, Ambrosio G, Milei J. Are kinking and coiling of carotid artery congenital or acquired? Angiology. 2010;61(1):107-12. <https://doi.org/10.1177/0003319709336417>.

49. Sacco S, Totaro R, Baldassarre M, Carolei A. Morphological variations of the internal carotid artery: Prevalence, characteristics and association with cerebrovascular disease. Int J Angiol. 2007;16(2):59-61. <https://doi.org/10.1055/s-0031-1278249>.

50. Togay-Işikay C, Kim J, Betterman K, Andrews C, Meads D, Tesh P, Tegeler C, Oztuna D. Carotid artery tortuosity, kinking, coiling: stroke risk factor, marker, or curiosity? Acta Neurol Belg. 2005;105(2):68-72.

51. Paulsen F, Tillmann B, Christofides C, Richter W, Koebke J. Curving and looping of the internal carotid artery in relation to the pharynx: frequency, embryology and clinical implications. J Anat. 2000;197 Pt 3:373-81. <https://doi.org/10.1046/j.1469-7580.2000.19730373.x>.

52. Pellegrino L, Prencipe G, Vairo F. Dolicho-arteriopathies (kinking, coiling, tortuoosity) of the carotid arteries: study by color doppler ultrasonography. Minerva Cardioangiol. 1998;46.

53. Del Corso L, Moruzzo D, Conte B, Agelli M, Romanelli AM, Pastine F, Protti M, Pentimone F, Baggiani G. Tortuosity, kinking, and coiling of the carotid artery: expression of atherosclerosis or aging? Angiology. 1998;49(5):361-71. <https://doi.org/10.1177/000331979804900505>.

54. Macchi C, Gulisano M, Giannelli F, Catini C, Pratesi C, Pacini P. Kinking of the human internal carotid artery: a statistical study in 100 healthy subjects by echocolor Doppler. J Cardiovasc Surg (Torino). 1997;38(6):629-37.

55. Oliviero U, Cocozza M, Picano T, Policino S, Russo N, Fazio S, Coto V, Saccá L. Prevalence of Carotid Kinking and Coiling in a Population at Risk. Vascular Surgery. 1997;31(1):43-9. <https://doi.org/10.1177/153857449703100106>.

56. Borioni R, Garofalo M, Actis Dato GM, Pierri MD, Caprara E, Albano P, Chiariello L. Kinking of internal carotid artery: is it a risk factor for cerebro-vascular damage in patients undergoing cardiac surgery? J Cardiovasc Surg (Torino). 1994;35(4):325-6.

57. Barbour PJ, Castaldo JE, Rae-Grant AD, Gee W, Reed JF, Jenny D, Longennecker J. Internal carotid artery redundancy is significantly associated with dissection. Stroke. 1994;25(6):1201-6. <https://doi.org/doi:10.1161/01.STR.25.6.1201>.

58. Metz H, Bannister RG, Murray-Leslie RM, Bull JWD, Marshall J. KINKING OF THE INTERNAL CAROTID ARTERY: in Relation to Cerebrovascular Disease. The Lancet. 1961;277(7174):424-6. <https://doi.org/https://doi.org/10.1016/S0140-6736(61)90004-6>.
